# Telemedicine Applications in the Era of COVID-19: Telesurgery Issues

**DOI:** 10.3390/ijerph19010323

**Published:** 2021-12-29

**Authors:** Paolo Bailo, Filippo Gibelli, Alberto Blandino, Andrea Piccinini, Giovanna Ricci, Ascanio Sirignano, Riccardo Zoja

**Affiliations:** 1 Section of Legal Medicine, School of Law, University of Camerino, 62032 Camerino, Italy; filippogibellimedico@gmail.com (F.G.); giovanna.ricci@unicam.it (G.R.); ascanio.sirignano@unicam.it (A.S.); 2 Sezione di Medicina Legale e delle Assicurazioni, Dipartimento di Scienze Biomediche per la Salute, Università degli Studi di Milano, Via Luigi Mangiagalli, 37, 20133 Milano, Italy; alberto.blandino@unimi.it (A.B.); andrea.piccinini@unimi.it (A.P.); riccardo.zoia@unimi.it (R.Z.)

**Keywords:** Telemedicine, Telesurgery, latency, malpractice, legal implications

## Abstract

Telemedicine allows for the effective delivery of health care to patients at a distance through the application of information technology to the field of medicine. This is optimal during the COVID-19 pandemic to reduce interpersonal contact to mitigate contagion. Among the possible Telemedicine applications, there is Telesurgery, which involves more and more surgical specialties thanks to the numerous benefits in quality and cost containment. In the growing field of Telesurgery, its technical and legal implications must be considered. In this study, a traditional review of the scientific literature was carried out to identify the most relevant issues of interest in Telesurgery. The problematic legal aspects identified are mainly related to the difference in legislation between different geographical areas, which is critical in the case of malpractice. In addition, there is the possibility of a malicious hacker attack on the transmitted data stream either to steal sensitive data or to harm the patient. Finally, there are inherent difficulties with the technology used, such as latency issues in data transmission. All these critical issues are currently not adequately addressed by current legislation. Therefore, one can only hope for a legislative action to allow Telesurgery to be used safely.

## 1. Introduction

The WHO defined Telemedicine as “*The delivery of health care services, where distance is a critical factor, by all health care professionals using information and communication technologies for the exchange of valid information for the diagnosis, treatment, and prevention of disease and injuries, research and evaluation, and for the continuing education of health care providers, all in the interests of advancing the health of individuals and their communities*” [1].

With the outbreak of the COVID-19 pandemic, public health policies have been implemented by the national governments that focus on the reduction of viral transmission: especially social distancing, with quarantine and isolation strategies [2]. In this context, Telemedicine has been chosen as an ideal tool to answer the challenge and face the emergency [3,4]. Thus, the introduction of Telemedicine into the healthcare landscape, being a new discipline not yet rooted in the territory, has opened the discussion regarding its legal, ethical, and regulatory implications [5]. Nittari et al. highlighted the issues present in this field, especially the lack of well-defined regulations and ethical issues regarding protecting patients’ data and informed consent [6].

A growing branch of Telemedicine is Telesurgery, which is defined as the use of medical technology, such as robotics, sensory devices, and imaging video that allows a surgeon to operate long distances [7]. Robotic-assisted surgery has been successfully used in urology, general surgery, gynecology, neurosurgery, and cardiothoracic surgery [8]. The main advantages of Telesurgery are the ability to perform immediate surgeries in remote regions while avoiding long-distance travel, maintaining high quality and low cost, and the consequent reduction in surgical staff required to meet patients’ demands [9]. In addition, it can be used for the training of new surgeons, known as telementoring [10].

The main objective of this traditional review is to describe state of the art in a rapidly growing field of telemedicine, telesurgery. Specifically, we propose an overview of telesurgery’s technical, legal, and privacy-related issues and suggest possible solutions. A secondary objective of our work is to present potential future applications of telesurgery to highlight its growing role in the healthcare landscape.

## 2. Telesurgery: Birth and Development 

Telesurgery was born in the laboratories of the US military and only later transferred to the civilian sector. The first applications were on the battlefields and on US Navy ships in the 1990s. Many laparoscopic operations were performed by a robot controlled by a surgeon at the Landsthul military hospital in Germany, 4000 km away [11]. The first remote surgery was carried out in 1995 in Milan by urologist Enrico Pisani, who performed a prostate biopsy on a patient located at the hospital, 5 km away from the surgeon, who was operating from the Polytechnic building [12].

Apart from this short-range pioneering experience, the first telesurgical operation took place on 7 September 2001. Professor Jacques Marescaux and his team from IRCAD (Institute for Research into Cancer of the Digestive System) performed a laparoscopic cholecystectomy operation by commanding from New York the mechanical arms of a robot surgeon located more than 6000 km away, in Strasbourg (France). A high-speed fiber-optic service provided the link between the robotic system and the surgeon [13]. The surgical procedure, named “Operation Lindbergh” in honor of Charles Lindbergh, the aviator who made the first non-stop flight across the Atlantic in 1927, was a milestone in modern medicine and represented the beginning of a new era.

Since then, there has been a continuous refinement of the technology behind Telesurgery, leading in 2014 to VIP (Virtual Interactive Presence) and in 2015 to the prototype that implemented efficiently haptic feedback technology, the Telefap Alf-x. In 2019, the use of Telesurgery via a 5G network was successfully tested. Today, we have a technical and scientific background that makes it possible to imagine a near future in which Telesurgery will be the norm.

## 3. Telemedicine Applications: More Than Just Telesurgery

Telemedicine applications are countless and can be applied to virtually any medical specialization, not just surgery. The most straightforward application is the so-called Televisit. It is a communication between doctor and patient mediated by a digital interface. Through Televisit, not only does the doctor have the possibility of conducting a dialogue with the patient to acquire as complete an anamnesis as possible, but he also has the possibility of subjecting the patient to, for example, cognitive tests. A recent study on Alzheimer’s disease patients showed that the reliability of clinical questionnaires, such as the MMSE (Mini-Mental State Examination) and the Alzheimer’s Disease Assessment Scale cognitive subscale (ADAS-cog) administered via teleconferencing is comparable to that of the same questionnaires administered face-to-face [14].

Teleradiology uses systems capable of acquiring, processing, transmitting, and archiving X-ray images in analog and digital formats. This application dates back to 1967 when a radiologist at Massachusetts General Hospital installed a diagnostic station at Boston’s Logan Airport. Doctors in transit brought images of their patients, which were video transmitted by the diaphanoscope, to the hospital to obtain the medical opinion of local radiologists. The audiovisual microwave circuit linking the hospital with the airport enabled more than 1000 medical consultations to be carried out for the benefit of airport employees and travelers [15]. The main advantage of Teleradiology is the possibility of telediagnosis, i.e., the remote interpretation of radiological images, which is particularly useful in conditions of particular environmental discomfort or when a radiologist’s opinion with specific expertise in a specific field is required. Telediagnosis is complemented by teleconsultation, i.e., two or more specialists can discuss the interpretation of particularly complex radiological images [16]. The possibility of teleconsultation is significant in modern medicine, where super-specialty branches are becoming increasingly common.

Telepathology is the histopathological transposition of teleradiology, where images of microscopic preparations replace radiological images. The microscopic image is captured by a camera, which then digitizes, compresses, and transmits it [17].

Telecardiology consists of the transmission of images obtainable from cardiology diagnostic equipment [18]. The most important field of application is the telereferral of electrocardiograms: an EKG carried out physically in one place is examined and interpreted by a cardiologist in another place. Another interesting area of application of Telecardiology that is gradually gaining ground is telerehabilitation. Cardiac telerehabilitation makes it possible to carry out all the exercises usually envisaged in cardiac rehabilitation, even in centers remotely connected to the cardiologist, where only the presence of nurses or physiotherapists is required. A third key area where Telemedicine applied to cardiology is highly beneficial is cardiac telemonitoring. Patients in need of cardiac monitoring can use wristwatches, wireless watches, and event recorders with finger electrodes to record their EKG traces from the comfort of their own homes and share them with their doctor.

The shortage of healthcare staff, particularly pharmacists, has meant that measures have had to be taken to implement drug distribution services in recent years. Telepharmacy aims to provide an effective solution to this problem. Telepharmacy services are based on the presence of three types of structures operating in a network: the coordinating pharmacy, i.e., the reference pharmacy, is managed by a pharmacist; the remote pharmacy, i.e., the peripheral pharmacy, is driven by a pharmacy technician; the remote dispensing site, i.e., the vending kiosk located inside clinics or other buildings.

The remote pharmacy and dispensing site are linked to the coordinating pharmacy via an audio-video computer system. There are essentially three pharmaceutical practices in which Telepharmacy plays a leading role: support to clinical services, remote education and handling of “special pharmacies,” and prescription and reconciliation of drug therapies [19].

There is also Tele-assistance and Tele-rescue [20]. Tele-assistance is a form of medical support provided remotely to a patient, directly at home (tele-homecare). It is a particularly suitable tool for periodically checking a patient’s general conditions and intercepting and managing any emergencies requiring direct medical intervention. Remote assistance is a service mainly aimed at the elderly, the disabled, those living alone or in a state of isolation. It is also beneficial for those working in particularly isolated and hard-to-reach environments, such as seafarers [21]. It allows people in an emergency or distressing situation to request immediate medical assistance by simply operating a device directly connected to an assistance center.

## 4. The Privacy Issues

Confidentiality and the consequent management of patient information are of crucial importance. The data collected and transmitted during the treatment are sensitive information. It is possible to know the patient’s health status related to the type of operation carried out and by observing the organs outside the surgical procedure but affected by the surgical field or further diagnostic procedures [22]. For example, if it is necessary to perform ultrasound procedures that may show tumors, malformations, or sexual gender, security measures must be optimal. Therefore, since the data is electronic, it is imperative to have adequate computer security systems in place.

Nonetheless, the patient must be fully informed about the type of data collected, how it will be transmitted, and how it will be stored. In addition, it must be specified where and for how long this data will be held. All this follows the current legislation of the countries where the procedure is performed [23].

## 5. Technical Issues: Latency Time, Depth Perception and Tactile Perception

Unlike Telemedicine, which, even though it takes place in a relatively short period, does not require a continuous and stable flow of data, Telesurgery can only be performed in real-time and with a connection that guarantees adequate data transmission speed and stability [24]. A signal that is not fast enough can give false information to the surgeon, who is operating at a distance, and force him to make choices that are not in keeping with the actual situation, causing not only a delay in the operation with poor management of any emergencies, such as bleeding, but also incorrect maneuvers that can cause potentially severe or fatal injuries [8].

The first studies carried out with the advent of the telesurgical technique have questioned the problem of finding the optimal latency, defined as the time of delay in data transfer, above which you should never go to avoid intraoperative complications [25]. For a better definition, *“signal latency refers to the amount of time it takes an input from the physician’s console to reach the robot or, conversely, the speed at which the signal travels from the cameras to the physician, allowing the physician to see the structures”* [26].

During the first approaches to the telesurgical technique through exercises carried out without patients, surgeons had determined that the minimum latency to operate safely should be less than 330 milliseconds (ms) [13]. Further studies [27,28] determined that only latency up to 200 ms can be considered acceptable. Still, Korte et al. found that a delay of more than 2 s causes inefficiency and inaccuracy, regardless of operator experience, further emphasizing the vital importance of this variable [29]. According to Wirz et al., a latency time greater than 300 milliseconds produces significant inaccuracies in instrument handling. The ideal latency time should be less than 100 ms [30]. These results were confirmed by further latency tests showing that the surgeon begins to feel a delay in his movements at 300 ms; from 300 to 500 ms, the surgeon can slow down his movements to compensate for the delay, stopping after each action. Above 600 ms, it is no longer possible to operate safely [31].

The institution offering the Telesurgery service must be able to maintain an optimal connection. This feature is feasible in several areas where the relationship is well established (e.g., USA) and with the possibility of 5G connectivity systems. Still, it is problematic in less developed regions [32]. Moreover, the longer the intervention lasts, the greater the possibility of connection instability with possible consequent damage to the patient [33]. 

At the moment, the aspect of latency is not regulated by any regulation or law. From a liability point of view, it is necessary to identify the subject who could be required to compensate for damages: liability profiles could be directed towards the entity offering the procedure, the provider of the data transmission service, and the software producer. This is also necessary to be able to provide and have adequate insurance cover.

Regarding purely operational aspects, one of the most apparent problems with Telesurgery is that the operator does not directly view the surgical field, operating through a screen interface. This is an issue that affects remote surgery and the whole field of laparoscopic surgery. Fortunately, there are several strategies to obtain 3D information from endoscopic images.

The two most widely used techniques are optical coherence tomography, which can render two-dimensional images three-dimensionally, and the use of the 3D endoscope, which allows three-dimensional viewing by using a pair of optical channels to generate two images of the same site but viewed from a slightly different angle (reproducing the retinal disparity that ensures stereoscopic vision) [34]. The drawback of using 3D endoscopes is the need for the operator to wear special glasses with polarization filters, which only allow light to pass through in the same polarization mode, separating the image for the right and left eye. The use of these glasses has proven to be highly disturbing for operators, as it can cause side effects, such as nausea, dizziness, and lightheadedness. A solution to the problem of using 3D glasses has come with developing autostereoscopic displays [35]. These are displays with a special technology (e.g., the lenticular system) that can hide the image intended for the other eye independently for each eye, without the need for external media. The only limitations of autostereoscopic displays are a relatively narrow viewing angle and low resolution due to the spatial multiplex technology.

Another significant operational issue is the lack of tactile perception by the surgeon operating the robot from a distance. The technology that enables the transmission of tactile information to the teleoperator is termed “haptic feedback.” Examples of elementary-level haptic feedback are joysticks with force feedback and mice where the wheel locks when the pointer reaches the edge of the screen. Since the dawn of Telesurgery, systems have been designed to allow the surgeon to perceive tactile sensations directly due to his maneuvers.

The first Telesurgery prototype equipped with an efficient haptic feedback system was Telelap Alf-x, presented in 2015 [36]. In addition to providing the surgeon with precise haptic feedback, the system was also equipped with eye-tracking technology, which could stop robotic arm movements when the operator’s eyes were not fixed on the screen. The system has been shown to reduce operating times significantly (e.g., a cholecystectomy was shortened by 60 min, from 90 to 30 min) [37].

## 6. New Perspectives: The 5G Network

Fifth-generation (5G) wireless networks enable significantly more stable data transmission than their predecessors and up to 100 times faster (10 GB/s). Virtually no signal latency can be achieved by reducing latency to a surprisingly low 1–2 ms. The application of 5G technology to telematics is extraordinarily promising.

In December 2020, an Italian research team published the results of the first intervention performed remotely using a robot connected to a 5G network [38]. The intervention consisted of a transoral laser microsurgery procedure performed on a cadaver. In a room 15 km away from the anatomy laboratory of the San Raffaele Hospital in Milan, where the corpse was located, the surgeon removed a polyp artificially grafted onto the body’s vocal cords. An experimental 5G network provided two-way data transmission linking both ends of the system (the robot surgeon and the human surgeon). The intervention went smoothly, with no connection problems. 

A more recent study published in Jama Ophthalmology attempted to assess the real-world viability of laser photocoagulation for diabetic retinopathy performed remotely, in real-time, through the support of 5G technology [39]. The study involved an ophthalmic surgeon from Peking Union Medical College Hospital in Beijing, China, who performed the procedure on six patients (nine eyes) between October 2019 and July 2020. The patients were examined by teleconsultation and then underwent Telesurgery through a laser system and a remote-control platform connected to a 5G network. From a technical point of view, with an average network latency time of 20 milliseconds, the surgeries were conducted without any delay affecting the surgical action. No loss of image quality or signal loss was detected. This is one of the first studies to assess the reliability of 5G in its application to Telesurgery, and further randomized clinical trials are needed before widespread application can be envisaged.

In a recent paper, a group of Chinese surgeons reported the results of 12 spinal surgeries performed via telesurgery with a 5G network. Throughout 12 operations, the surgeons implanted 62 pedicle screws. The average latency time of the interventions was 28 milliseconds; no disconnection events were recorded. The operations were completed without any intraoperative complications. This further proves the promising nature of applying 5G technology to telesurgery [40].

## 7. Legal Issues 

Telesurgery has immediately introduced new legal issues. Medical malpractice in the operating room is directly related to the work of the surgeon. With the advent of Telesurgery, one must consider that the operator will no longer interface directly with the patient but through other instrumentation that may introduce substantial risks. This fact increases the possible sources of liability involved in the event of any injury to the patient. In addition, the increase in the number of services due to greater accessibility by patients to medical services can only increase the risk of malpractice [41].

The geographic distance between patient and physician is a significant feature of Telemedicine and Telesurgery. This aspect opens up legal scenarios that have not yet been well explored and defined, e.g., damage due to latency—as previously discussed—and the possibility of performing a healthcare service between different nations or territories with different legislative regulations [42]. The legislation of one state may be in contrast with the legislation of another state, creating a conflict of laws. In this regard, many situations can be created. One possible concern is that if a healthcare professional licensed to work exclusively in his or her territory performs services in a different region, he or she is committing an illegal act. Additionally, if the professional commits an error during the procedure, which legislation must respond and in which court? In this context, countless scenarios can develop in that an intervention may be legal in one country and not in another, or the legislation of one country may be more favorable than that of another [43].

The problems highlighted find their most significant expression in the courtroom where proceedings are initiated. The court is called to decide in which jurisdiction to operate, and in case it succeeds in making a choice, it can apply the reference legislation. Still, all this is extremely difficult also because of the possible objections between the parties involved [44]. Currently, there is no international legislation regulating the legal aspects of Telesurgery, but this is also lacking in individual countries. 

There are several possible interpretations of the doctor-patient relationship: the most widely accepted is that the legislation to be applied is that of the state where the patient is located [45]. 

Others maintain that it is as if the patient were “transported” by the doctor during the operation. Therefore, it is necessary to apply the legislation of the state where the doctor works. However, the latter interpretation is not supported by the law [46].

Other legal issues are the need to certify and validate telesurgical networks and have unique lists of physicians qualified to perform this type of service [47]. Telesurgical networks can be attacked by malicious parties, both for data theft and to create malfunctions that result in patient injury. It is possible to use encryption of transmitted data and a data flow monitoring system to make telesurgical procedures more secure. However, these measures do not entirely exclude the risk of malicious data streams and compromising the robotic console from the very beginning [48]. For these reasons, a standard should be defined.

Then there is the crucial matter of medical professional liability. In the case of telesurgery, the legal issues do not differ substantially from those of ordinary medical liability. The fundamental element of distinction is represented by the fact that telesurgery implies that the surgeon’s conduct is filtered and processed by a computerized machine. Thus, the source of any error with detrimental consequences for the patient’s health may lie with the person who initiates the maneuver (the surgeon) or how the conduct is carried out (the machinery). Therefore, impeccable surgical actions may result in technical errors due to bugs, blockages, maintenance defects of the machinery. Thus, when assessing damages related to the use of telesurgery, it is essential to evaluate as accurately as possible the causal link between the conduct and the event to correctly interpret its genesis and define the division of responsibilities between man and machine. Suppose the technical error was to be traced back to a defect in the machinery through which the surgeon exercises his technical action. In that case, it seems rather evident that the manufacturer or the programmer would be held responsible (alone or jointly with the physician, depending on the situation). 

An entirely different situation arises in the case of so-called “robot surgeons,” i.e., robots equipped with artificial intelligence sophisticated enough to enable them to perform operations autonomously. In 2016, researchers at the Children’s National Health System in Washington DC designed a robot that could autonomously suture a pig intestine [49]. It is evident that when artificial intelligence is introduced, it is not always easy to attribute any error in the robot to the manufacturer, the programmer, or the certifying body. This is because robots’ behavior, based on automatic learning modes that are difficult to control, involves a certain amount of unpredictability. If, however, an unexpected outcome were to be attributed to the programmer, foreseeability should be understood, in very general terms, as referring to future damage that cannot be precisely identified beforehand (therefore, not referring to damage produced during the intervention). This perspective, however, seems difficult to accept in terms of the universally understood principle of guilt [50].

Another interesting issue—more deontological than legal—raised by telesurgery is accessibility to healthcare. The principle that every human being has the right to have access to the best care is enshrined in the most important international treaties: the Universal Declaration of Human Rights (Art. 25), the Constitution of the World Health Organization (Art. 1), the European Social Charter (Art. 11), the International Covenant on Economic, Social and Cultural Rights (Art. 12).

This is a principle of justice, according to which unprejudiced access to care and equitable distribution of technological resources to support medical progress must be universally pursued. The technological breakthrough provided by telemedicine and telesurgery could represent a restriction of access to healthcare for patients located in socio-culturally backward realities (due to lack of access to the Internet, smartphones, or other technologies) [51]. To resolve this sensitive issue, the practice of telemedicine and telesurgery requires precise and codified rules of conduct at the international level.

An interesting attempt in this direction was recently made by an American research group, which organized a workshop with key stakeholders in rural telesurgery. The stakeholders were surgeons, technology experts, and representatives of the telemedicine world (lawyers, economists, regulatory consultants, administrators of hospitals in rural locations, nurses, and payers). At the end of the workshop, the researchers identified four main topics of interest (operating room team interactions, education and training, network and security, and economic issues). On these bases, they prepared an operational plan to make telesurgery available in rural settings [52].

## 8. Conclusions

Due to technological development and the pandemic situation, Telemedicine has spread rapidly, allowing increased access to care. In addition, Telesurgery can help with surgical procedures performed in the current pandemic: minimizing surgical staff in operating rooms has allowed the risk of COVID-19 infection to be lowered [53]. The use of these new technologies has brought to light several issues, including legal ones.

In this review, we have highlighted the main problems related to the legal aspects of the telesurgical branch of Telemedicine. The highlighted panorama shows that, despite the rapid development of this technique due to its numerous application possibilities, the required legislative support is totally lacking and inadequate. The main challenges concern formulating laws related to the IT component that surgeons have to interface with data privacy management. Nonetheless, it is essential to overcome the problem of territorial attribution of existing legislation among different states.

National governments should address these deficiencies by addressing the problem and creating appropriate legislation. Failure to do so could result in technologically advanced procedures that expose patients to serious health risks and little chance of compensation.

The main limitation of our work lies in the design of the study. The presented topic is unevenly addressed in the current literature mainly because of its novelty and rapid development. Therefore, instead of conducting a scoping review, we preferred to perform a traditional literature review, which is helpful to provide a general and wide-ranging view of a specific topic, less accurate but still solid and of interest.

## Data Availability

Not applicable.
